# Unveiling the Integral Role of Magnetic Resonance Imaging in the Comprehensive Evaluation and Diagnosis of Spinal Dysraphism

**DOI:** 10.7759/cureus.60972

**Published:** 2024-05-24

**Authors:** Ravi Kumar Yeli, Dhanya S B, Sunil H C, Gowthami G S, Srikalyan Duddukuri, Praveen Kumar M

**Affiliations:** 1 Department of Radiology, Bijapur Lingayat District Educational (BLDE) (Deemed to be University) Shri B. M. Patil Medical College Hospital and Research Centre, Vijayapur, IND; 2 Department of Radiology, Jagadguru Sri Shivarathreeshwara (JSS) Medical College, Mysuru, IND; 3 Department of Radiology, Kanachur Institute of Medical Sciences, Mangalore, IND; 4 Department of Paediatrics, Al-Ameen Medical College and Hospital, Vijayapur, IND; 5 Department of Radiology, Asian Institute of Gastroenterology (AIG) Hospital, Hyderabad, IND; 6 Department of Radiology, Sri Jayadeva Institute of Cardiovascular Sciences and Research, Mysore, IND

**Keywords:** tissue differentiation, mri, non-invasive imaging, spinal dysraphism, congenital anomalies

## Abstract

Background

Spinal dysraphism, characterized by incomplete closure of neural and bone spinal structures, manifests as congenital fusion abnormalities along the dorsal midline, involving the skin, subcutaneous tissue, meninges, vertebrae, and neural tissue. Magnetic resonance imaging (MRI), the preferred imaging modality for assessing spinal dysraphism across all age groups, provides direct visualization of the spinal cord without the need for contrast or ionizing radiation while also eliminating bone artifacts and allowing multiplanar imaging. The objective of this study was to evaluate the range of spinal dysraphism lesions and assess the significance of MRI in their evaluation.

Methodology

Thirty patients with suspected spinal dysraphism underwent evaluation at the Medical College Hospital and Study Centre in Vijayapur, India. This cross-sectional observational study included patients diagnosed or provisionally diagnosed with spinal dysraphism based on clinical and imaging profiles. Cases were identified through preliminary findings on radiographs.

Results

The study encompassed individuals aged one month to 20 years, with the largest proportion of patients (36.67%) falling within the 1-5-year age group. Spina bifida was the most prevalent spinal abnormality, accounting for 70% of cases. In 12 patients (40%), the most prevalent location of involvement was the lumbosacral spine.

Conclusion

MRI provides excellent tissue differentiation, particularly of lipomatous tissue, with reproducible and comprehensive section planes and relative operator independence. Moreover, MRI is beneficial for children with suspected spinal dysraphism as it can be performed without ionizing radiation, biological risks, or the need for intrathecal contrast media.

## Introduction

Spinal dysraphism encompasses a range of congenital anomalies involving the fusion of dorsal midline tissues, including the bone, connective tissue, and nervous system tissue [1,2]. Among others, Lichtenstein [3] and James and Lassman [4] provided the first description of it. Computed tomography (CT), ultrasonography, and conventional radiography were all used in traditional imaging techniques [5,6]. According to Barnes et al., the first magnetic resonance imaging (MRI) of spinal dysraphism was described [7].

In around one to three out of every 1,000 live infants, spinal dysraphism is a prevalent congenital disease with substantial rates of death and morbidity [8]. Females account for a notable proportion (55-70%) of neural tube abnormalities. Location, gender, race, and ethnicity are some of the variables that affect prevalence [9,10]. Prenatal screening and folic acid supplementation have contributed to a reduction in the frequency of neural tube abnormalities during the past 25 years [10]. The majority of instances of closed spinal dysraphism are asymptomatic at birth, but they may be suspected if there are high-risk cutaneous indicators or if the infant develops neurological problems later on [11]. MRI is the recommended evaluation method because of its great soft tissue detail, better imaging capabilities, and usefulness for preoperative planning [8]. Anteroposterior and lateral plain radiographs are essential for evaluating the vertebral column [12,13]. Bony spurs may be seen in diastematomyelia instances, directing further imaging investigations. Ultrasonography is helpful for newborns and infants and can identify spinal dysraphism antenatally; however, because of posterior element ossification, its use decreases after the first year [14,15].

The open neural arch, flaring laminae, meningomyelocele sac, hydrocephalus, and related cranial abnormalities can all be detected by prenatal ultrasonography. Nevertheless, MRI is frequently necessary to obtain comprehensive, in-depth information. Bony spurs in situations of split cord malformation can be visualized using CT. The ideal methodology for assessing spinal dysraphism is MRI because of its accuracy and non-invasive nature. Its wide field of view, high-contrast resolution, and multiplanar imaging capabilities allow for the identification of cord tethering and related syringomyelia and a comprehensive assessment of the spinal cord and related back mass [13,16].

T2-weighted images help demonstrate syrinx and related disorders such as dermoid and epidermoid cysts. Multiple spinal abnormalities, such as syringohydromyelia or myelomeningocele with accompanying Chiari malformation, can coexist in patients with spinal dysraphism. When diagnosing spinal defects and associated hydrocephalus in utero, fetal MRI can be used in addition to ultrasonography [14,15].

Therefore, the study aims to assess the spectrum of spinal dysraphism lesions and determine the significance of MRI in evaluation. Through comprehensive analysis, the goal is to understand the manifestations, prognostic factors, and clinical implications of spinal dysraphism. Additionally, highlighting the efficacy of MRI in accurately diagnosing and characterizing these lesions is sought, with the ultimate goal of refining diagnostic and management strategies for affected individuals.

## Materials and methods

Study design and participants

In the Department of Radiology at the Shri B. M. Patil Medical College Hospital and Research Centre, Vijayapur, India, 30 patients who were suspected of having spinal dysraphism based on a clinical examination were included in the current observational study. Consent was obtained or waived by all participants in this study. The Institutional Ethical Committee of Shri B. M. Patil Medical College Hospital and Research Centre approved the study (approval number: IEC/Ret/100-142/17).

*Selection* *Criteria*

This study included patients diagnosed or provisionally diagnosed with spinal dysraphism, regardless of age or gender, based on clinical and imaging profiles. Patients were identified through preliminary findings on radiographs or incidentally detected cases on radiographs.

Data sources and variables

The study adhered to ethical guidelines, ensuring that permission was acquired from all patients or their legal guardians after providing comprehensive information about the study procedures. In cases of clinically suspected spinal dysraphism, a thorough diagnostic approach was employed. Ancillary radiological investigations encompassed a range of modalities tailored to the specific clinical presentation. This included comprehensive whole spine X-rays featuring anteroposterior and lateral views, with additional posteroanterior views for cases presenting with sizable back masses. Furthermore, oblique views were obtained to scrutinize suspected vertebral anomalies.

In instances of low-suspicion cases with potential bony defects, high-resolution ultrasonography served as an initial diagnostic tool. However, when the findings from plain radiographs and MRI scans were deemed inadequate, CT scans were employed for further evaluation. Notably, a Philips Brilliance 40-slice multi-detector CT scanner (Block Imaging, Holt, MI, USA) was utilized to ensure optimal imaging quality and diagnostic accuracy.

For a comprehensive assessment and detailed anatomical visualization, an MRI was conducted using a state-of-the-art 1.5-T SIEMENS magnetom (Siemens Healthineers, Erlangen, Germany). This imaging protocol included a spectrum of sequences such as T1 and T2 spin echo, T2 fluid-attenuated inversion recovery (FLAIR), short tau inversion recovery (STIR), gradient echo (GRE), and specialized chord sequences. This comprehensive approach aimed to facilitate precise diagnosis and management planning for patients with suspected spinal dysraphism, ensuring the highest standards of care and diagnostic accuracy.

Statistical analysis

Statistical analysis, encompassing both descriptive and inferential methods, was employed to analyze the data. Continuous measures are presented as mean±standard deviation (min-max), providing a comprehensive overview of the central tendency and variability within the dataset. Meanwhile, categorical measurements are depicted as numbers and percentages (%), offering insights into the distribution and prevalence of various categorical variables among the study population. 

## Results

Table 1 displays the distribution of patients under investigation across different age ranges. In this study, patients ranged in age from infancy to older childhood, with a total of 30 patients assessed. The largest group, accounting for 36.67% of patients, was between one and five years old, while 33.33% were over five years old. The youngest age group, from birth to one year, made up 30% of the study population.

**Table 1:** Age range of the patients under investigation

Age in years	Number of patients	Percentage
0-1 year	9	30%
1-5 years	11	36.67%
>5 years	10	33.33%
Total	30	100%

Table 2 presents the distribution of patient gender under investigation. This study analyzed a total of 30 patients across different age groups and genders. Among the female patients, the majority (52.94%) were aged between one and five years, with 29.41% in the 0-1-year age range and 17.64% over five years old. In total, females made up 56.67% of the study population. For the male patients, the highest proportion (55.6%) were over five years old, while 23.1% were aged between one and five years, and 12.5% were in the 0-1-year age group. Males comprised 43.33% of the total study population.

**Table 2:** Patient gender distribution under investigation The data has been presented in the form N (%)

Gender	Age in years			Total
	0-1 year	1-5 years	>5 years	
Female	5 (29.41%)	9 (52.94%)	3 (17.64%)	17 (56.67%)
Male	2 (12.5%)	7 (23.1%)	4 (55.6%)	13 (43.33%)
Total	7 (100%)	16 (100%)	7 (100%)	30 (100%)

Table 3 illustrates the distribution of cutaneous lesions among the patients under investigation. The study investigated the distribution of cutaneous lesions among patients. The most common clinical feature was swelling in the back, observed in 14 patients, which accounted for 46.67% of the cases. Urinary incontinence and dermal sinus each affected five patients, making up 16.67%, respectively. Lower limb weakness was seen in three patients (10%), while sacral dimple was noted in two patients (6.67%). Hypertrichosis was the least common feature, affecting only one patient (3.33%).

**Table 3:** Distribution of patients' cutaneous lesions under investigation

Sr. no.	Clinical features	Number	Percentage (%)
1	Swelling in back	14	46.67%
2	Hypertrichosis	1	3.33%
3	Sacral dimple	2	6.67%
4	Lower limb weakness	3	10%
5	Urinary incontinence	5	16.67%
6	Dermal sinus	5	16.67%

Table 4 delineates the variations in the distribution of spinal dysraphism types according to the age range of the individuals under investigation. The study assessed the distribution of different types of spinal dysraphism across various age groups among the 30 patients studied. Spina bifida was the most common type, affecting 21 patients (70%). This included seven patients (33.33%) in the 0-1-year age group, eight patients (38.10%) in the 1-5-year age group, and six patients (28.57%) over five years old. 

**Table 4:** Variations in the distribution of spinal dysraphism types according to the age range of the individuals under investigation The data has been presented in the form N (%)

Variables	Age in years			Total (n=30)
	0-1 year (n=9)	1-5 years (n=11)	>5 years (n=10)	
Spina bifida	7 (33.33%)	8 (38.10%)	6 (28.57%)	21 (70%)
Myelomeningocele	4 (50%)	2 (25%)	2 (25%)	8 (26.7%)
Myelocele	1 (12.5%)	1 (7.7%)	0 (0%)	2 (6.7%)
Lipomyelomeningocele	2 (20%)	6 (60)	2 (20%)	10 (33.33%)
Lipomyelocele	0 (0%)	1 (100%)	0 (0%)	1 (3.33%)
Diastematomyelia	3 (33.33%)	3 (33.33%)	3 (33.33%)	9 (30%)

Table 5 illustrates the variations in the distribution of spinal dysraphism types based on the age range of the individuals under investigation. The study examined the distribution of different types of spinal dysraphism across various age groups among 30 patients. The most common types of spinal dysraphism observed were tethered cord and syrinx.

**Table 5:** Variations in the distribution of spinal dysraphism types according to the age range of the individuals under investigation The data has been presented in the form N (%)

Variables	Age in years			Total (n=30)
	0-1 year (n=9)	1-5 years (n=11)	>5 years (n=10)	
Filar lipoma	0 (0%)	3 (60%)	2 (40%)	5 (16.67%)
Dorsal dermal sinus	1 (25%)	2 (50%)	1 (25%)	4 (13.33%)
Syrinx	5 (25%)	11 (55%)	3 (15%)	20 (66.67%)
Tethered cord	7 (31.89%)	8 (36.37%)	7 (31.89%)	22 (73.33%)
Sacral agenesis	0 (0%)	4 (57.14%)	3 (42.86%)	7 (23.33%)
Vertebral segmentation anomalies	3 (27.27%)	6 (54.55%)	2 (18.18%)	11 (36.67%)
Scoliosis	4 (23.53%)	9 (52.94%)	4 (23.53%)	17 (56.67%)

Table 6 presents the examination of the distribution of patients' sites of involvement. The study analyzed the site of involvement among 30 patients with spinal dysraphism. The most commonly affected areas were the lumbosacral and lumbar regions. The lumbosacral region was involved in 12 patients, accounting for 40% of the cases. The lumbar region was affected in nine patients, making up 30% of the cases. 

**Table 6:** The distribution of patients' sites of involvement was examined

Sr. no.	Site involvement	Number	Percentage (%)
1	Cervical	2	6.67
2	Thoracic	3	10
3	Dorsolumbar	2	6.67
4	Lumbar	9	30
5	Lumbosacral	12	40
6	Sacral	2	6.67

Table 7 demonstrates the dispersion of Arnold-Chiari malformation type II (ACM-II) in connection with patient age distribution. The study assessed the distribution of ACM-II across various age groups among 30 patients. Out of the total population, ACM-II was found in eight patients (26.67%). 

**Table 7:** Dispersion of ACM-II in connection to patient age dispersion The data has been presented in the form N (%) ACM-II: Arnold-Chiari malformation type II

ACM-II	Age in years			Total
	0-1 year	1-5 years	>5 years	
Negative	5 (50%)	10 (83.3%)	7 (87.5%)	22 (73.3%)
Positive	5 (50%)	2 (16.67%)	1 (12.5%)	8 (26.67%)
Total	10 (100%)	12 (100%)	8 (100%)	30 (100%)

## Discussion

Spinal dysraphism refers to congenital anomalies of the spine and spinal cord. This study assessed the role of MRI in evaluating suspected spinal dysraphism in patients aged one month to 20 years. Most patients (36.67%) were between the ages of one and five years, consistent with prior studies, such as Kumari et al. [8] and Nafees et al. [17], where the majority of patients were under five years old. In this study, of the 30 patients examined, 10 (33.33%) had open spinal dysraphism, while 20 (66.66%) had closed spinal dysraphism. This distribution differed from Kumari et al.'s [8] study, which found 42.4% of patients with closed dysraphism and 57.5% with open dysraphism. Similar findings were reported in the study by Hosagavi et al. [18].

Clinical signs of spinal dysraphism in children can include back swelling, dermal sinus, hemangioma, dimples, lower limb weakness, and difficulties with bowel and bladder control. In this study, back swelling was the most common clinical feature, affecting 14 individuals (46.67%). Kumari et al. also noted back swelling as the most prevalent clinical characteristic, affecting 77.2% of patients [8].

Among the various forms of dysraphism, lipomyelomeningocele emerged as the predominant type of closed spinal dysraphism, impacting 10 patients (33.33%). Myelomeningocele was the most frequent type of open spinal dysraphism, involving eight patients (26.6%). Kumari et al. [8] also reported that 57.5% of patients had myelomeningocele, while Nafees et al. [17] found that the majority of their patients (39.2%) had this condition. Studies by Hosagavi et al. [18] and Vohra et al. [19] also found myelomeningocele to be the most common type of open spinal dysraphism.

In terms of location, among the eight patients with myelomeningocele in this study, one was in the cervical region, one was in the lumbar region, one was in the sacral region, and five were in the lumbosacral region. Kumari et al. [8] and Nafees et al. [17] reported that most patients with myelomeningocele had involvement in the lumbosacral area. Nine patients (33.3%) had diastematomyelia in this study, with 40% being type 2 and 60% type 1. Kumari et al. [8] reported a higher proportion of type 2 diastematomyelia (75%), while Nishtar et al. [20] found a lower incidence of diastematomyelia (4%).

Open defects often coexist with conditions such as syrinx, hydrocephalus, and ACM. In this study, ACM-II was found in eight patients (26.67%). Kumari et al. [8] reported it in 15.7% of patients, while Kumar and Singh [21] found it in 45% of patients. Overall, the study underscores the importance of MRI in diagnosing and assessing spinal dysraphism, providing crucial insights into the prevalence, types, and associated conditions of the disease.

Limitations of the study

The study's limitations include its small sample size of 30 patients and focus on individuals aged one month to 20 years, potentially limiting generalizability. Retrospective design could introduce bias, and variations in open/closed dysraphism cases compared to previous studies suggest demographic differences. Reliance on clinical signs and MRI may miss subtle cases, and long-term outcomes or treatment responses were not explored. MRI availability and cost may limit widespread use. Despite limitations, the study provides insights into MRI's role in spinal dysraphism evaluation, highlighting the need for cautious interpretation and future research.

## Conclusions

The diagnosis of spinal dysraphism, a complicated illness with several underlying causes that might present variably on imaging, is mostly dependent on MRI. MRI is quite good at differentiating tissue, especially lipomatous tissue, and it provides slice planes that are thorough and repeatable. Moreover, it is a safe choice for patients of all ages because of its non-invasiveness and lack of ionizing radiation. This cutting-edge imaging method is crucial for accurately detecting the spectrum of findings in patients with spinal dysraphism and for doing a comprehensive evaluation of the spinal cord.
